# The Arabian Seas: a window into future oceans —physical, biogeochemical, and ecological dynamics in unique marginal environments

**DOI:** 10.1093/nsr/nwag607

**Published:** 2026-09-23

**Authors:** Peng Zhan, Guoxin Cui, Daquan Guo, Song He, John A Gittings, Yannis Hatzonikolakis, Malika Kheireddine, George Krokos, Zouhair Lachkar, Zhiqiang Liu, Jingyi Ma, Jithendra Nadimpalli, Marianthi Pateraki, Dionysios E Raitsos, Dionysia Rigatou, Junchuan Sun, Panagiotis Vasou, Yixin Wang, Yiling Zheng, Ibrahim Hoteit

**Affiliations:** Department of Ocean Science and Engineering, Southern University of Science and Technology, Shenzhen 518055, China; Division of Physical Science and Engineering, King Abdullah University of Science and Technology (KAUST), Thuwal 23955-6900, Saudi Arabia; Division of Physical Science and Engineering, King Abdullah University of Science and Technology (KAUST), Thuwal 23955-6900, Saudi Arabia; Simon F.S. Li Marine Science Laboratory, School of Life Sciences, The Chinese University of Hong Kong, Hong Kong 999077, China; Division of Physical Science and Engineering, King Abdullah University of Science and Technology (KAUST), Thuwal 23955-6900, Saudi Arabia; Department of Ocean Science and Engineering, Southern University of Science and Technology, Shenzhen 518055, China; Division of Physical Science and Engineering, King Abdullah University of Science and Technology (KAUST), Thuwal 23955-6900, Saudi Arabia; Department of Biology, National and Kapodistrian University of Athens, Athens 15771, Greece; Division of Physical Science and Engineering, King Abdullah University of Science and Technology (KAUST), Thuwal 23955-6900, Saudi Arabia; Center for remote sensing applications, Mohammed VI Polytechnic University, Ben Guerir 43150, Morocco; Division of Physical Science and Engineering, King Abdullah University of Science and Technology (KAUST), Thuwal 23955-6900, Saudi Arabia; Mubadala Arabian Center for Climate and Environmental Sciences (ACCESS), NYUAD Research Institute, NYU Abu Dhabi, Abu Dhabi 129188, UAE; Department of Ocean Science and Engineering, Southern University of Science and Technology, Shenzhen 518055, China; Center for Complex Flows and Soft Matter Research, Southern University of Science and Technology, Shenzhen 518055, China; Division of Physical Science and Engineering, King Abdullah University of Science and Technology (KAUST), Thuwal 23955-6900, Saudi Arabia; Division of Physical Science and Engineering, King Abdullah University of Science and Technology (KAUST), Thuwal 23955-6900, Saudi Arabia; Division of Physical Science and Engineering, King Abdullah University of Science and Technology (KAUST), Thuwal 23955-6900, Saudi Arabia; Department of Biology, National and Kapodistrian University of Athens, Athens 15771, Greece; Department of Biology, National and Kapodistrian University of Athens, Athens 15771, Greece; Division of Physical Science and Engineering, King Abdullah University of Science and Technology (KAUST), Thuwal 23955-6900, Saudi Arabia; Division of Physical Science and Engineering, King Abdullah University of Science and Technology (KAUST), Thuwal 23955-6900, Saudi Arabia; Division of Physical Science and Engineering, King Abdullah University of Science and Technology (KAUST), Thuwal 23955-6900, Saudi Arabia; Division of Physical Science and Engineering, King Abdullah University of Science and Technology (KAUST), Thuwal 23955-6900, Saudi Arabia; Division of Physical Science and Engineering, King Abdullah University of Science and Technology (KAUST), Thuwal 23955-6900, Saudi Arabia

**Keywords:** Arabian Seas, planetary boundaries, future oceans, marine ecosystems

## Abstract

The Arabian Sea provide a sentinel system for understanding how global planetary boundary transgressions manifest through regional ocean processes. Their rapid warming, altered circulation, declining ventilation, deoxygenation, acidification, and ecological reorganization reveal interconnected regional manifestations of pressures associated with the Climate Change, Biogeochemical Flows, Ocean Acidification, and Biosphere Integrity boundaries. Regional physical and biogeochemical amplification compresses the timescale over which these pressures become ecologically consequential, exposing regional responses and increasing risks of regime shifts. Although the Arabian Seas possess distinctive environmental characteristics, the mechanisms linking planetary forcing to regional change are broadly relevant to other tropical and marginal oceans. Their present trajectory therefore provides a conditional, mechanism-based analogue for selected processes expected to intensify across future tropical open-ocean and marginal-sea environments. This illustrates how multiple global boundary transgressions may interact to weaken regional resilience and reorganize marine ecosystem function.

## Teaser

The Arabian Seas are already experiencing combinations of warming, oxygen loss, acidification and ecological change that may become more widespread in future oceans. Their rapidly changing environments reveal how global climate pressures can be amplified by regional ocean processes, reshaping marine ecosystems and testing the limits of their resilience.

## INTRODUCTION

The Arabian Seas, comprising the Arabian Sea (AS) in the northwestern Indian Ocean, the Red Sea (RS), and the Arabian Gulf (AG), represent one of the most dynamic marine environments on Earth. Under monsoon forcing, seasonal upwelling, and extreme evaporative regimes with limited river discharges [1,2], the region has been recognized as a theater of extremes: It hosts one of the world’s most energetic boundary current systems [3], some of the warmest and saltiest surface waters [4], a mid-depth oxygen minimum zone (OMZ) of marked intensity [5], and ecosystems that have adapted to such extreme conditions [6].

However, in an era of rapid anthropogenic change, the scientific significance of the Arabian Seas has transcended regional oceanography. It has emerged as a critical window into the future oceans where the environmental conditions predicted for tropical marine ecosystems are already a present-day reality. The role of the Arabian Seas as a natural laboratory stems from the existence of multiple climate stressors that are rarely observed together elsewhere. For instance, the RS, with its rapid warming rates, serves as a testbed for thermal adaptation [6]; the AG, a shallow evaporation basin, offers a glimpse into a hyper-saline, ultra-warm future [7]; and the open AS demonstrates the consequences of deoxygenation on pelagic productivity near the physiological limits of marine viability [8]. By examining how these coupled physical-biogeochemical systems operate under present-day extremes, the Arabian Seas provide a conditional analogue for future ocean conditions. Here, we do not propose that the present Arabian Seas reproduce the future mean physical or ecological state of the global tropical ocean. Rather, the region allows mechanisms expected to intensify under anthropogenic forcing to be investigated under present-day high-stressor conditions. The open AS provides the principal basis for comparison with future tropical open-ocean processes, whereas the RS and AG provide extreme analogs for warm and evaporative marginal seas.

These environmental stresses make the framework of planetary boundaries (PBs) particularly relevant. The concept of PB defines safe operating limits for key Earth system processes by identifying nine critical Earth system processes, beyond which the planet’s stable state could be disrupted [9,10]. Recent assessments reveal that six of these nine global boundaries are already transgressed, pushing the Earth system well outside the Holocene-like window of environmental variability [11]. The translation of these global PB transgressions to regional scales, however, is not a simple linear downscaling [12]. While we do not propose arbitrary regional thresholds or standalone Arabian Seas boundaries, it is imperative to analyze how global control variables manifest locally [11]. In the Arabian Seas, ocean warming under climate change is amplified in the semi-enclosed seas; intense evaporation pushes the freshwater cycle to its limits; nutrient cycling imbalances manifest as anoxic dead zones, and cumulative anthropogenic pressures are increasing rapidly [13,14]. These observable phenomena represent regional manifestations of pressures associated with global PB transgressions.

This review is structured around the cascading interactions of four out of the nine PBs: Climate Change, Biogeochemical Flows, Ocean Acidification, and Biosphere Integrity. We first examine the response to the Climate Change boundary by analyzing regional hydrodynamics, which sets the stage to investigate regional manifestations of pressures associated with the Biogeochemical Flows and Ocean Acidification boundaries. These processes then culminate in the changes to Biosphere Integrity, where we evaluate ecosystem reorganization and the testing of physiological resilience from coral reefs to pelagic food webs. Beyond global forcing alone, direct anthropogenic pressures modify both environmental exposure and ecosystem resilience. The Arabian Seas serve as a present-day sentinel for multiple physical and ecological mechanisms likely to intensify across tropical oceans and marginal seas under continuous climate change. Those mechanisms of adaptation provide an essential blueprint for the adaptive governance and observation strategies required to navigate the Anthropocene globally.

## PHYSICAL AMPLIFIERS OF CLIMATE STRESS

The physical constraints of the Arabian Seas, including semi-enclosed geometries, hyper-aridity, extreme warmth, and hypersalinity, accelerate physical and biogeochemical responses to climate forcing. Therefore, processes projected for many tropical marine ecosystems are already observable in the Arabian Seas. By acting as physical amplifiers, these localized dynamics translate large-scale climate perturbations into immediate ecosystem stressors, making the regional response an indicator of broader future ocean change.

### Circulation and vulnerable pathways

The Arabian Seas are experiencing increasing thermal stratification and changes in ventilation superimposed on their strong seasonal circulation [15]. This transition provides an example of how climate warming may weaken ventilation in tropical and marginal oceans more broadly. The physical architecture of the AS is governed by a vigorous monsoon-driven surface circulation coupled with the buoyancy-driven overturning of its marginal basins [3]. During the boreal summer (southwest monsoon), topographically steered winds, notably the Findlater Jet, drive the Somali Current northward as one of the world’s most energetic boundary currents [16,17]. This intense forcing generates the Great Whirl (GW) alongside the retroflection of the East African Coastal Current (EACC) in the western basin [1,18]. In winter, the northeast monsoon drives the West India Coastal Current (WICC) poleward in the eastern basin, fueled by convective cooling and remote forcing via coastal Kelvin waves [3,19]. However, continued warming and stronger upper-ocean stratification are expected to modify this predictable seasonal pattern, leading to weakening of vertical exchange and mid-depth ventilation [7].

The intermediate depths of the AS are ventilated primarily by Indian Ocean Central Water [20], while high-salinity outflows originating in the marginal seas, the Arabian Gulf Water (AGW) and the RS Outflow Water (RSOW), provide important regional lateral exchange (Fig. 1a) [7,21]. AGW exits through the Strait of Hormuz, and typically equilibrates at *∼*150–300 m, near the upper boundary of the OMZ, where it contributes to ventilation of the oxycline [22]. In contrast, RSOW descends through the Strait of Bab-el-Mandeb and spreads mainly at depths of *∼*600–1000 m, undergoing substantial mixing and transformation in the Gulf of Aden before entering the western AS [23]. Although its oxygen contribution is less well constrained than that of AGW, RSOW may provide additional ventilation to deeper western AS [24]. Its influence depends strongly on the volume and properties of the exported water and on the substantial mixing and transformation that occur in the Gulf of Aden [25].

**Figure 1.**
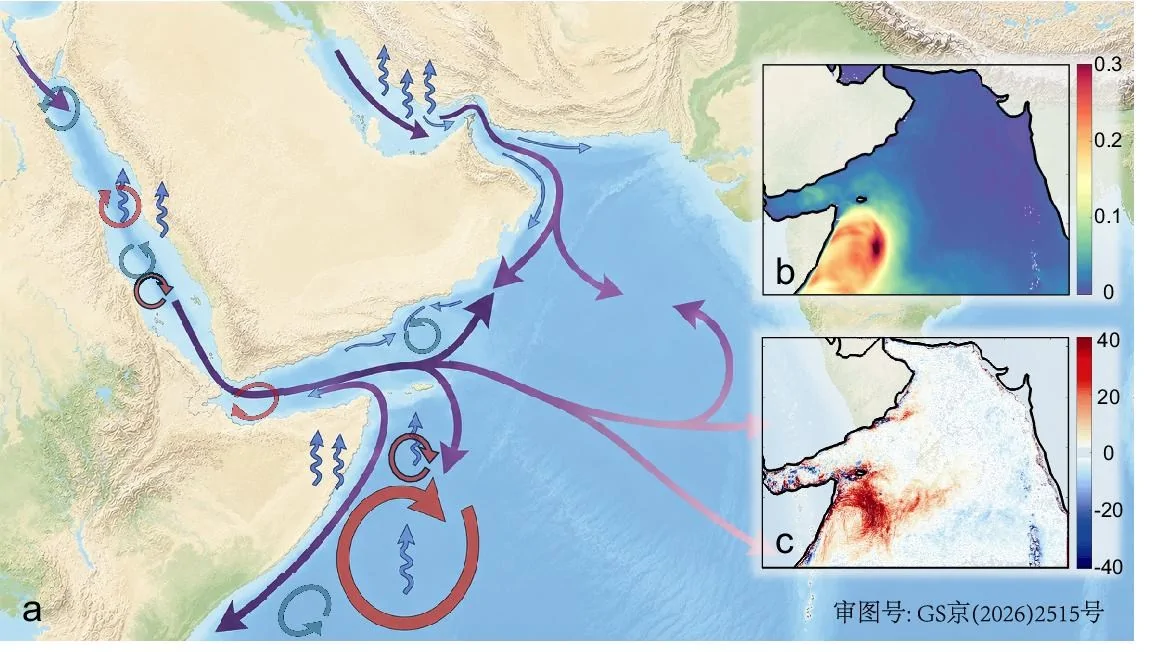
(a) Map illustrating key oceanographic features in the Arabian Seas. Broad pathways indicate the subsurface spreading of high-salinity Red Sea Outflow Water (RSOW) from the Bab-el-Mandeb Strait and Arabian Gulf Water (AGW) from the Strait of Hormuz. Gradual changes in the pathway shading represent dilution as these water masses spread into the open Arabian Sea. Circular arrows indicate anticyclonic (clockwise) and cyclonic (counterclockwise) mesoscale eddies, including the prominent Great Whirl off the Somali coast. Upward-pointing wavy arrows indicate regions of significant airsea heat exchange. (b) Mesoscale eddy kinetic energy in summer ($\mathrm{m^2\, s^{-2}}$). (c) Submesoscale vertical heat flux at 50 m in summer ($\mathrm{W\, m^{-2}}$), with positive and negative values corresponding to upward and downward heat flux, respectively.

However, continued warming may weaken marginal-sea ventilation by reducing source-water density and overturning strength. For RSOW, warming and reduced renewal within the RS could produce lighter and less oxygenated exported waters, although the downstream consequences remain insufficiently quantified. High-resolution eddy-resolving simulations demonstrate that under a $4^{\circ }$C AG warming scenario, the mean potential density of the AGW decreases from *26.5± 0.1* to *25.7± 0.1*  $\rm {kg\, m}^{-3}$ [25]. This resulting increase in buoyancy limits the capacity of the outflow to cascade down the continental slope, causing the AGW to equilibrate at much shallower depths (100–150 m) [25].

Recent observational estimates suggest that AGW provides a critical oxygen contribution to the AS OMZ of *∼*1.3 $\rm {Tmol\, yr}^{-1}$ [5]. The projected shoaling of AGW under warming would reduce the volume of outflow reaching intermediate depths, weaken its contact with the OMZ, and diminish its contribution to the upper-OMZ ventilation [25]. Separately, observations indicate a long-term expansion of the OMZ, with the volume of the suboxic core expanding by *> *50% since the 1970s [26]. Concurrently, rapid upper-ocean warming has strengthened stratification and reduced winter convective mixing, particularly in the northern AS [27]. These processes may reduce the supply of oxygen to the intermediate layers and contribute to the expansion of the OMZ’s suboxic core, thereby amplifying the regional expression of the transgression of the global Biogeochemical Flows PB [25,28]. The sensitivity of these marginal sea ventilation pathways has broader relevance for understanding climate-driven changes in other tropical and marginal oceans. Localized warming within marginal seas and at their restricted gateways can alter source-water formation, outflow density, and downstream penetration, with consequences for downstream deoxygenation and ecological restructuring in the open ocean [5,28]. In the Arabian Seas, these effects reflect the combined evolution of monsoon circulation, marginal-sea overturning, and intermediate-depth transport rather than changes in a single ventilation pathway.

### Mesoscale eddies and submesoscale fronts

In an increasingly stratified future ocean, multi-scale turbulent mixing driven by eddies and submesoscale processes represents a critical, yet highly vulnerable, physical pathway for vertical nutrient and oxygen fluxes.

Beyond large-scale circulation, the Arabian Seas are dynamically structured by highly energetic mesoscale eddies $(O(100)\, \rm {km})$ [29,30] and submesoscale processes $(O(1-10)\, \rm {km})$ that mediate the vertical and lateral fluxes of heat, salt, nutrients, and oxygen [31,32]. The eddy fields exhibit strong regional contrasts in their phenology. The western AS is a global hotspot for mesoscale eddy kinetic energy (EKE) during the summer (Fig. 1b), driven by potent baroclinic and barotropic instabilities stemming from monsoon-driven coastal upwelling and the Findlater Jet [30,33]. In contrast, the RS exhibits winter-peaking submesoscale and mesoscale activities, particularly in the central and northern basins, driven by intense surface buoyancy loss, frontogenesis, and mixed-layer instability (MLI) [34,35]. Horizontally, these eddies are essential for the transport and spreading of the RSOW and AGW, redistributing thermohaline and biogeochemical signatures throughout the open ocean [36]. Vertically, mesoscale strains and frontal instabilities generate high-frequency vertical motions, penetrating intense stratification gradients and effectively pumping nutrients from the aphotic interior to the surface while ventilating intermediate layers [37,38].

Studies of highly energetic boundary currents, such as the Kuroshio Extension, demonstrate that traditional satellite altimetry systematically underestimates EKE by *∼*40% because it fails to adequately resolve submesoscale filaments and frontal instabilities [39]. We infer a similar observational gap exists in the Arabian Seas, as high-resolution models reveal that submesoscale vertical heat fluxes (SVHF) can reach up to 80 $\text{ W m}^{-2}$ during winter in the northern RS, a magnitude comparable to global hotspots like the Kuroshio Extension [35]. In the western AS, strong SVHF during the summer monsoon are comparable to air–sea heat fluxes (Fig. 1c), and are at least twice as large as the mesoscale vertical heat fluxes (MVHF) [40]. Meanwhile, strong SVHF amplify MVHF to create a cross-scale positive feedback loop that maintains sharp cross-shore buoyancy gradients and drive upward heat transport from the subsurface [41].

The same ageostrophic secondary circulations that generate vertical heat transport can also transport nutrients and oxygen. During the winter bloom period, the vertical eddy flow accounts for *∼*40% of the nitrate in the upper layer, with this proportion increasing to *∼*90% during the summer bloom period [20]. Marine ecosystems in the Arabian Seas rely heavily on mixing-driven nutrient supply to sustain the functional component of Biosphere Integrity. However, the system faces uncertainty under the transgression of global Climate Change PB. As warming intensifies stratification, the resulting restriction of diapycnal mixing creates a biogeochemical bottleneck. It remains uncertain whether submesoscale frontogenesis acts as a resilience mechanism by injecting nutrient and oxygen pulses into the euphotic zone to offset stratification [38,42], or as an accelerator of ecosystem collapse, whereby submesoscale restratification further suppresses deeper diapycnal nutrient supply [43]. Regardless, the intensification of these multi-scale dynamics under altered wind forcing directly modulates the stoichiometric imbalances of the upper ocean, dictating the spatial limits of the euphotic nutrient pump [32,44].

### Internal wave dissipation and diapycnal mixing

While wind-driven circulation dominates upper-ocean energetics, internal wave dissipation and baroclinic tides govern vertical diffusivity and diapycnal mixing in the deep interior and along continental margins [45], which is important to sustain biological productivity. Across the Arabian Seas, baroclinic tides and internal waves act as the primary physical drivers of diapycnal mixing. In the open AS, the internal tide field is characterized by persistent, highly energetic mode-1 $M_2$ beams radiating from prominent topographic generation sites, including Socotra Island, the Carlsberg Ridge, the Maldives, and the northeastern AS shelf, though they are frequently refracted and deflected by the monsoon-driven mesoscale eddy field (Fig. 2) [46]. This enhances vertical diffusivity along beam intersections and rough continental slopes, driving episodic ventilation into the subsurface layers [47]. The energetics of these baroclinic tides are modulated by stratification and therefore display pronounced seasonality, with winter domain-integrated barotropic-to-baroclinic conversion rate *∼*50% higher than that in summer [48].

**Figure 2.**
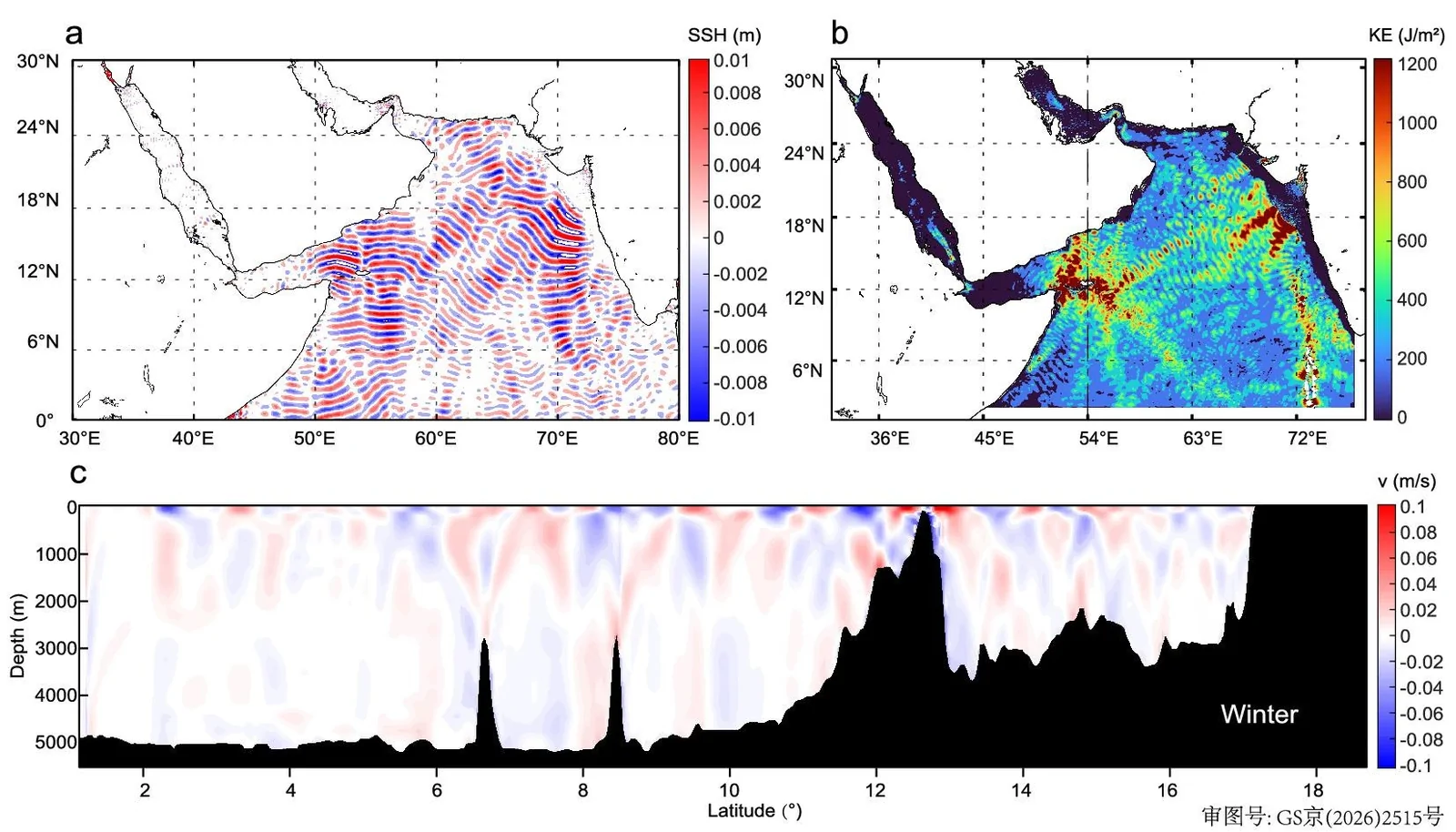
(a) Snapshot of mode-1 $M_2$ internal-tide SSH at 00:53:20 on 5 November 2015, derived from multi-altimeter observations. (b) Kinetic energy of internal tides in January from the simulation. The dashed lines indicate the locations of the transects shown in (c). (c) Snapshot of velocity along the cross-section at $54.68^\circ$E at 12:00 on 28 January 2011. Figure adapted from Fig. 3 of Ref. [46] and Figs 6 and 7 of Ref. [48].

In the marginal seas, steep bathymetry and distinct tidal forcing create highly localized mixing hotspots. In the southern RS, tide-topography interactions generate high-frequency internal solitary wave (ISW) packets with peak amplitudes exceeding 70 m and coherent crest lengths over 80 km [49]. The shoaling and breaking of these ISWs along the shelf slopes drive critical diapycnal mixing, representing a vital mechanism for benthic-pelagic connectivity that transports nutrients and larvae to shallow coral reef ecosystems [50]. Similarly, in the shallow AG, baroclinic tides peak during the summer, driving bottom-intensified turbulence and shear that resuspends sediments and periodically resets boundary layers [29].

The dynamics of internal wave mixing represent a critical physical resistance mechanism against the transgression of the global Biogeochemical Flows PB. Global internal tide dissipation over ridges and seamounts supplies up to 62% of the tidally generated nitrate flux into the euphotic zone [51]. At the OMZ boundary in the AS, enhanced diapycnal mixing physically reshapes the oxycline, directly modulating the nitrification-denitrification balances [52]. In the oligotrophic AG and RS, the breaking of internal waves re-fertilizes the lower euphotic zone and modulates the transport of larvae, providing a crucial connectivity mechanism between the deep benthos and shallow coral reefs [53].

Under the transgression of the global Climate Change PB, climate-driven thermal stratification and anomalous freshwater inputs from extreme precipitation events would alter vertical diapycnal diffusivity [45,54]. Observations in the Gulf of Oman indicate that intermittent shear-driven mixing prevails 14% of the time, enhancing double-diffusive salt fingering driven by the hypersaline AGW intrusions [5]. As climate change increases the buoyancy of these intrusions, the altered stratification suppresses this mechanical shear and double-diffusive mixing [24,55]. The future resilience of the region’s Biosphere Integrity depends heavily on whether global climate warming alters the internal energy conversion. While global projections indicate that enhanced stratification may theoretically funnel more energy into high-mode internal tides [56], if overall thermal stabilization successfully suppresses background mixing, the physical pumping of nutrients and oxygen will decline. This would reduce the physical supply of nutrients and oxygen supporting fisheries and reef ecosystems, illustrating how increasing stratification may elevate the risk of cascading biogeochemical and ecological impacts.

### Teleconnections as modulators of regional dynamics

Remote atmosphere–ocean teleconnections provide pathways through which global climate variability modulates regional marine conditions across the Arabian Seas (Fig. 3). On multi-decadal timescales, the Atlantic Multi-decadal Oscillation (AMO) strongly influences the regional thermal trajectory: *> *70% of multi-decadal AS sea surface temperature (SST) variability has been attributed to AMO-related trans-basin forcing [57], while RS SST is highly correlated with the AMO (*r=0.91*), with the regional response lagging the AMO by *∼*6 years [58]. On interannual timescales, the El Niño–Southern Oscillation (ENSO) and Indian Ocean Dipole (IOD) exert major controls on regional extremes [59]. El Niño modifies the Walker Circulation and midlatitude Rossby-wave trains, producing winter warming in the AS and reduced surface salinity in the RS [60]. Through the Indian Ocean Capacitor effect, this warming can persist into the following boreal summer, sustained by local air–sea feedbacks and downwelling oceanic Rossby waves [61]. The IOD additionally affects the Arabian Seas by modulating the local Hadley circulation and South Asian High [62]; during positive IOD events, SST anomalies in the central and western Arabian Gulf (AG) can reach $\sim 0.35^{\circ }\mathrm{C}$ [63]. Recent analyses further indicate that the combined effects of ENSO and the North Atlantic Oscillation (NAO) explain *> *50% of observed interannual summer SST variability in the AG [64].

**Figure 3.**
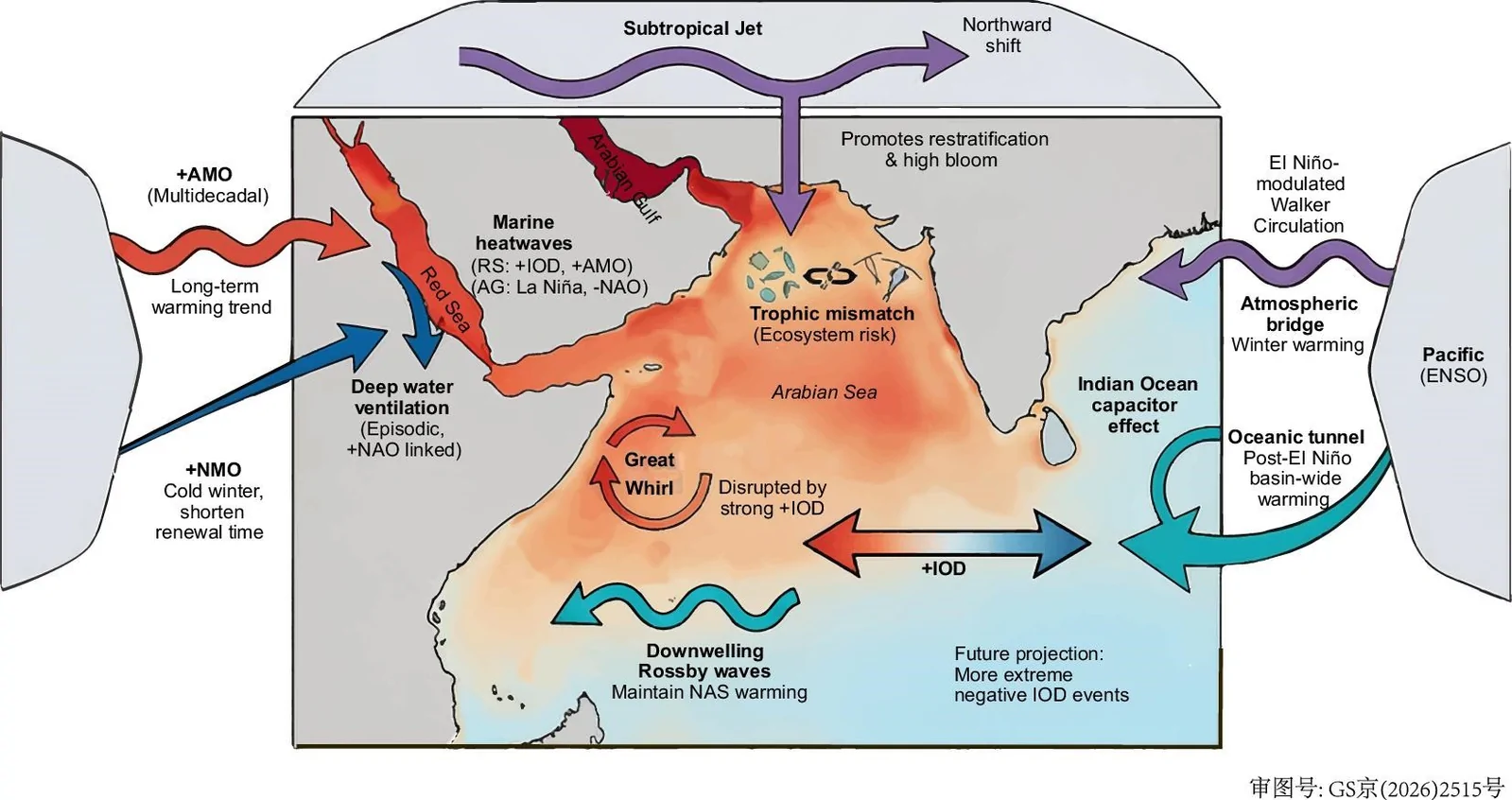
Schematic of climate teleconnections and physical processes governing the Arabian Seas. This diagram illustrates the complex network of remote atmospheric bridges and oceanic tunnels influencing the Arabian Seas. (1) Atlantic Forcing (left arrows): The positive AMO drives a long-term multi-decadal warming baseline. Positive NAO triggers episodic cold winters that are essential for RS deep-water ventilation. Negative NAO may inhibit renewal pulses, increasing the risk of deep-basin hypoxia. (2) Pacific and Indian Ocean Forcing (right and center arrows): El Niño modulates the Walker Circulation (atmospheric bridge), causing winter warming. Through the Indian Ocean Capacitor effect (oceanic tunnel), this warming is prolonged well into the summer, sustained by downwelling Rossby waves and local air–sea interactions. (3) Ecosystem Consequences (center): The convergence of these remote physical drivers can increase risks to Biosphere Integrity. Specifically, an anomalous northward shift of the Subtropical Jet accelerates mixed-layer restratification, triggering high-bloom scenarios in the northern basin. Concurrently, thermal anomalies precipitate intense Marine Heatwaves that push coral reefs toward their physiological limits, while suppressed vertical mixing, and circulation disruptions can increase the risk of trophic mismatch within the pelagic food web.

These teleconnections also affect regional biogeochemical processes. Episodic RS deep-water renewal is promoted by anomalously cold winters associated with the positive NAO [65]; conversely, negative-NAO conditions may suppress convective renewal, lengthen deep-water residence times, and increase the risk of deep-basin hypoxia. Large-scale climate variability also modulates the Subtropical Jet, with a northward jet displacement generating anomalous anticyclonic circulation and accelerating mixed-layer restratification, enhancing nutrient retention and favoring high-bloom conditions [66]. The IOD can further influence oxygen distributions. A coupled physical–biogeochemical simulation found that the suboxic area of the AS OMZ decreased by *∼*27% following the extreme 2016 negative IOD but expanded by *∼*28% following the extreme 2019 positive IOD [67]. These event-specific estimates illustrate how remote climate variability can propagate into regional biogeochemical responses.

Constraining the future ocean analogy is complicated by uncertainties in how the Indo-Pacific mean state and related Earth System Models (ESMs) will evolve. In historical simulations, Coupled Model Intercomparison Project Phase 6 (CMIP6) models often have mean-state biases that limit the representation of these teleconnections [68]. Common issues include equatorial Pacific cold tongue biases and mean-state positive IOD-like biases, leading to excessive westward extension of SST anomalies during ENSO and underestimated amplitude asymmetry of both ENSO and IOD [69,70]. Furthermore, observations show the tropical Pacific developing a stronger temperature contrast, characterized by a La Niña-like trend over recent decades, while most models fail to reproduce this historical trend [71]. These discrepancies introduce structural uncertainties into projections of the future variability of both ENSO and the IOD.

These uncertainties propagate into the physical and biogeochemical indicators of the Arabian Seas, complicating projections of regional response to global PB transgressions. Because ENSO and IOD phases strongly modulate basin-wide wind stress curl and Walker circulation subsidence, errors in their frequency and amplitude cascade into the assessment of stratification, mixed layer depth, vertical mixing, and cross-strait water exchange [68,72].

These physical discrepancies directly compromise regional biogeochemical projections. The physical ventilation of the AS OMZ relies heavily on the subduction of dense marginal sea outflows and turbulent mesoscale mixing [8,26]. Because coarse ESMs improperly represent teleconnection-driven natural variability, their estimates of intermediate-depth oxygen supply and OMZ sensitivity remain uncertain [73]. Consequently, determining whether the AS OMZ will expand due to restricted physical ventilation or contract due to biologically driven oxygen demand reductions relies heavily on accurately resolving these CMIP6 teleconnection biases. In addition, the synchronization of remote climate forcings with localized anthropogenic warming actively exacerbates the frequency and severity of extreme temperature events. This thermal stress pushes coral reef ecosystems closer to their physiological thermal limits. Therefore, the future trajectory of the Arabian Seas depends on a robust understanding of how the evolving mean state and climate modes will reshape the environmental thresholds governing regional ecosystem viability.

### Thermodynamic anomalies and hydrographic extremes

The Arabian Seas are undergoing rapid thermodynamic and hydrographic changes that outpace many global mean rates [74]. The AS is experiencing an accelerated SST warming rate of *∼*0.2$^\circ$C per decade [15,57]. The marginal seas exhibit even more extreme trajectories, with the RS warming at rates between 0.17$^\circ$C and 0.45$^\circ$C per decade [75,76], and the AG experiencing severe warming trends approaching *∼*0.6$^\circ$C per decade [25]. This rising thermal baseline has led to increasing Marine Heatwave (MHW) frequency, duration, and intensity. In the AS, MHW duration is increasing by *∼*20 days per decade since the 1980s [77]. The marginal seas amplify this trend with even greater severity; in the AG, MHW intensity is rising by $\sim 0.17^\circ$C per decade [77]. In the RS, 46% of all recorded MHW events and 58% of total MHW days over the last 40 years have occurred in the most recent decade alone [76].

As an evaporation-dominated basin, the AS is broadly consistent with the global tendency for salty regions to become saltier under an intensified hydrological cycle [78]. However, its upper-ocean salinity variability is strongly shaped by monsoon-driven circulation and lateral advection from adjacent water masses [79]. In contrast, the RS exhibits pronounced interannual surface-salinity extremes linked to ENSO variability [60]. Wintertime surface buoyancy loss, driven by net heat loss and evaporation-induced salinification, promotes convective mixing and intermediate-to-deep water formation [80]. In the northern AS, localized extreme precipitation events can enhance upper-ocean stratification, cap the mixed layer, and weaken AS High-Salinity Water formation [54]. In the AG, the compounding hazards of thermal stress and hypersalinity amplify the vulnerability of local ecosystems, forcing reef-building corals beyond their upper thermal and osmotic limits [4].

Importantly, interpreting these physical trends requires navigating controversies and uncertainties regarding how global climate modes propagate into the basin. Climate models struggle to resolve the narrow geometry of the Strait of Hormuz and the Tokar wind gap, leading to diverging projections for the AS [5,28]. While some coarse models predict a future contraction of the OMZ due to altered mean states and reduced biological export, higher-resolution models that permit finer-scale processes project an OMZ expansion and subsequent ecological reorganization [73,81]. Reducing these uncertainties is essential for improving projection capabilities of future tropical and marginal oceans.

## RESHAPING BIOGEOCHEMICAL PATHWAYS

The physical drivers discussed above directly influence the biogeochemical system of the Arabian Seas, reshaping nutrient pathways, oxygen distributions, and carbon cycling across seasonal to climate timescales. Nutrient cycling and productivity form the primary interface between circulation variability and ecosystem response, while oxygen dynamics and carbon transformations integrate the cumulative effects of ventilation, respiration, and air–sea exchange under ongoing climate change. Therefore, the Arabian Seas function as a sentinel system for understanding the future evolution of tropical marine ecosystems. The regional biogeochemical manifestations of global PB transgressions in the open AS offer highly transferable, broad lessons for the future of global open-ocean environments, while the RS and the AG offer more extreme scenarios due to their unique geometries, restricted exchanges, and hyper-evaporative regimes.

### Nutrient and productivity

The Arabian Seas exhibit contrasting responses in nutrient cycling under ongoing climate warming. Open basins are becoming increasingly oligotrophic, whereas coastal anthropogenic pressures are promoting eutrophication in shallow marginal seas. Productivity in the AS is constrained by nutrient availability, with seasonally reversing monsoon winds acting as the dominant physical forcing that regulates mixing, mesoscale circulation, and marginal-sea exchanges [82,83]. These processes mediate the vertical and lateral fluxes of essential nutrients, which are currently shifting under the climate change PB. As a result, the region offers an opportunity to examine how marine productivity responds to global environmental changes.

Productivity in the AS strongly depends on subsurface nutrient reservoirs and displays two distinct peaks associated with monsoon-driven physical mechanisms [84] (Fig. 4). During the southwest summer monsoon, productivity is fueled primarily by coastal upwelling, which is strongest in the western AS [82], whereas convective mixing during the northeast monsoon drives the winter productivity peak [83]. Horizontal advection and open-ocean mixing sustain productivity in the central AS [85]. Beyond these seasonal cycles, episodic events such as mesoscale eddies and tropical cyclones, as well as large-scale atmospheric circulation and decadal climate variability, can further modulate nutrient availability and productivity [83]. In the northern and central AS, iron and silicate often do not reach the surface because they are concentrated deeper [86]. While atmospheric deposition of aerosols can partially alleviate this limitation, it is insufficient and frequently leads to High-Nutrient–Low-Chlorophyll (HNLC) conditions [86].

**Figure 4.**
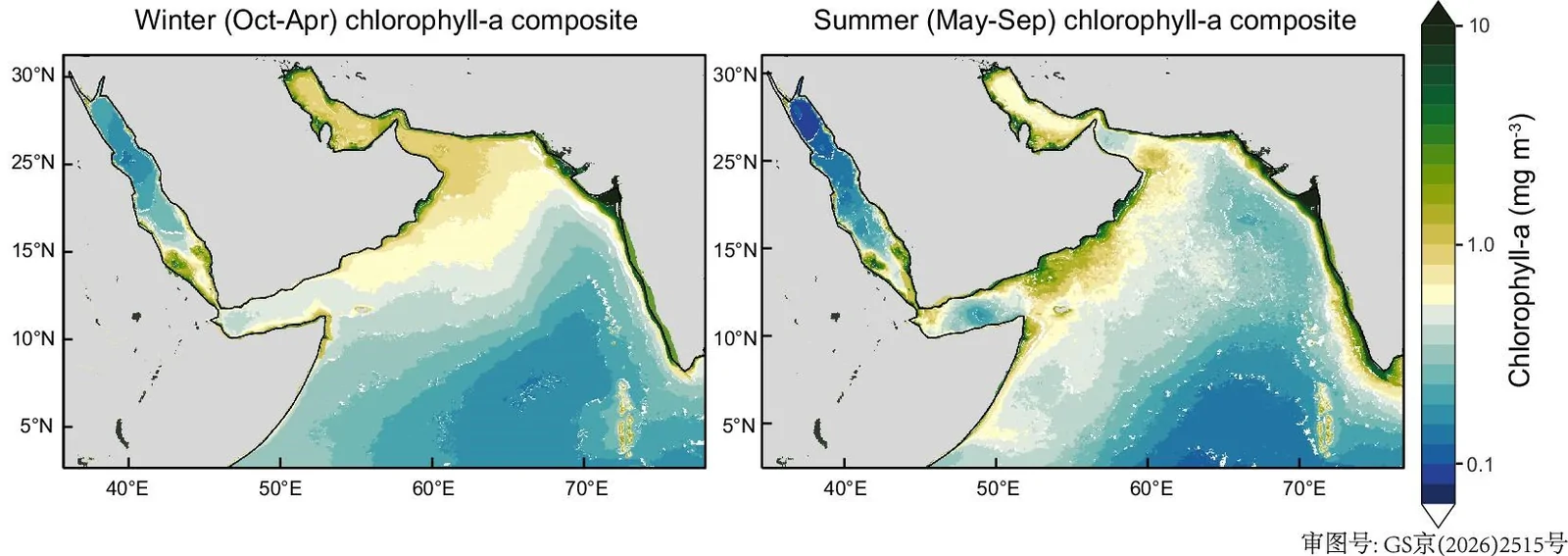
Spatial composites of chlorophyll—a concentration ($\rm {mg\,m}^{-3}$), a proxy for phytoplankton biomass, across the Arabian Seas. The left and right panels show the average chlorophyll—a distribution during winter (October–April) and summer (May–September), respectively. The influence of the monsoons on phytoplankton biomass distribution is evident, highlighting distinct seasonal patterns across the Arabian Seas. Chlorophyll—a data were obtained at a monthly temporal resolution from version 6.0 of the European Space Agency Ocean Colour Climate Change Initiative (ESA OC-CCI; https://climate.esa.int/en/projects/ocean-colour/).

In contrast to the AS, where natural biogeochemical cycling remains strongly modulated by climate forcing, the AG illustrates how human perturbations of biogeochemical flows dominate system dynamics. Under natural conditions, the AG exhibits strong basin-scale gradients in nutrients and productivity, with the northwestern basin displaying elevated phytoplankton biomass linked to nutrient-rich riverine discharge from the Shatt Al-Arab system [87]. Meanwhile, the southeastern basin receives nitrate-depleted Indian Ocean water, likely favoring diazotrophy [88]. However, rapid coastal development and untreated wastewater discharge have increased nutrient loads in the AG, fueling harmful algal blooms and hypoxia [89]. In a region already characterized by extreme temperatures and rising salinity due to river damming and high evaporation, anthropogenic nutrient enrichment interacts with these physical stressors and is likely to contribute to oxygen depletion and the expansion of coastal hypoxic zones [90].

The RS basin is highly oligotrophic, with minimal riverine or atmospheric inputs, so nutrient availability is largely controlled by the horizontal advection of nutrient-rich intermediate waters from the Gulf of Aden and their subsequent vertical redistribution by winter convection and mesoscale eddies [72]. Thus, the basin exhibits a non-linear north-south macronutrient gradient and is primarily nitrogen- and phosphorus-limited [91]. Dependence on this external supply generates a pronounced meridional gradient in phytoplankton biomass that declines from south to north [92] (Fig. 4), while mesoscale activity and episodic mixing locally impact this pattern [93]. The northern RS relies largely on winter deep convection for nutrient entrainment [94], whereas the south remains more productive and warmer year-round and can even experience harmful algal blooms [95].

However, the transgression of the global Biogeochemical Flows PB is altering key biogeochemical pathways in the Arabian Seas. The regional response can be measured by the volume of intermediate waters crossing the suboxic threshold, which dictates the transition from aerobic respiration to anaerobic microbial pathways, specifically denitrification [8,26]. The removal of bioavailable nitrogen (NO$_3^-$, NO$_2^-$, NH$_4^+$) decouples the nutrient stoichiometry essential for the productivity, creating a feedback loop that constrains the region’s biological carrying capacity. Water-column denitrification in the AS is estimated to range from 1 to 33 Tg N yr$^{-1}$, alongside significant benthic denitrification reaching up to 10.5 Tg N yr$^{-1}$ on the Pakistan margin [26]. Consequently, although the AS occupies *< *2% of the global ocean, it accounts for *∼*20%–40% of global pelagic nitrogen loss [25,73]. Furthermore, the byproduct of this anaerobic shift is the production of nitrous oxide (N$_2$O), a potent greenhouse gas that reinforces atmospheric warming, thereby creating a positive feedback loop between ocean deoxygenation and the climate system [96].

The altered physical-biogeochemical environment may drive ecological regime shifts and a reorganization of ecosystem structure and function [96]. In the northern AS, the traditional winter-bloom ecosystem, heavily dominated by diatoms, is being replaced by the mixotrophic dinoflagellate *Noctiluca scintillans* [97,98]. This ecological shift is triggered by a combination of physical and biogeochemical drivers. The loss of Eurasian snow cover has weakened winter convective mixing, leading to intensified upper-ocean stratification, while the expanding hypoxic boundary alters the N:P ratio via denitrification [27,97]. Diatoms, requiring high concentrations of inorganic nitrate and silicate, are outcompeted in this environment. Whether this community reorganization is reversible remains uncertain, because the relative roles of oxygen conditions, stratification, nutrient stoichiometry, and food-web feedbacks have not yet been resolved [8].

In addition, continued warming threatens the persistence of biogeochemical nutrient pathways. For instance, rapid warming strengthens stratification and suppresses the convection events that ventilate the deep basin and replenish surface nutrients [99]. Meanwhile, changes in the circulation at the Bab-el-Mandeb Strait may limit the inflow of nutrient-rich Gulf of Aden Intermediate Water. As the physical nutrient pump weakens, the system is increasingly forced to rely on regenerated production and rapid microbial recycling [100]. Such conditions are likely to restructure pelagic communities toward microbial-dominated assemblages, with implications for higher trophic levels in this enclosed basin.

### Oxygen budget and OMZ dynamics

The rapid deoxygenation of the Arabian Seas provides an example of biogeochemical threshold response, illustrating how global loading may push marine systems past the suboxic threshold, potentially into anaerobic regime shifts. The AS hosts the world’s thickest and most intense OMZ, occupying the intermediate depths from *∼*150–1250 meters, maintained by a delicate balance between physical oxygen supply and intense biological oxygen demand [26]. While this OMZ covers only roughly 2% of the global ocean surface area, it functions as a biogeochemical engine with disproportionate global influence, accounting for an estimated 10%–30% of total global pelagic denitrification [101].

Intense biological consumption, fueled by the monsoon-driven surface productivity and organic matter export detailed in preceding sections, depletes oxygen in the water column. Meanwhile, the semi-enclosed geometry and sluggish intermediate circulation restrict the lateral replenishment of oxygen. Ventilation of these intermediate waters is provided primarily by Indian Ocean Central Water, a blend of aged Subantarctic Mode Water and Indonesian Intermediate Water transported northward by the Somali Current [102]. However, secondary ventilation pathways driven by marginal sea outflows play a pivotal yet structurally vulnerable role in stabilizing the upper OMZ [73]. Underwater glider observations indicate that AGW supplies *∼*1.3 Tmol yr$^{-1}$ of oxygen to the AS OMZ with large interannual variability of *±*1.6 Tmol yr$^{-1}$ [5]. The RS and the AG act as regional lungs, with the denser RSOW sinking to *∼*600–1000 m and undergoing extensive mixing before reaching the core of the OMZ [36]. In contrast, the lighter AGW equilibrates at shallower depths of 150–300 m [36], playing a critical role in ventilating the oxycline and preventing the vertical expansion of suboxic waters into the euphotic zone [98]. Additionally, the diapycnal transport of this oxygen into the OMZ interior is modulated by fine-scale turbulence. Intermittent mechanical shear prevails 14% of the time at the oxycline, amplifying double-diffusive salt fingering and driving critical vertical oxygen fluxes into the suboxic core [5,7]. While global ocean oxygen inventories have declined by *∼*2% in recent decades [103], the AS is experiencing a more acute trajectory. Observations indicate a substantial intensification of the OMZ over the past six decades, with the volume of the suboxic core expanding by *> *50% since the 1970s [26] (Fig. 5).

**Figure 5.**
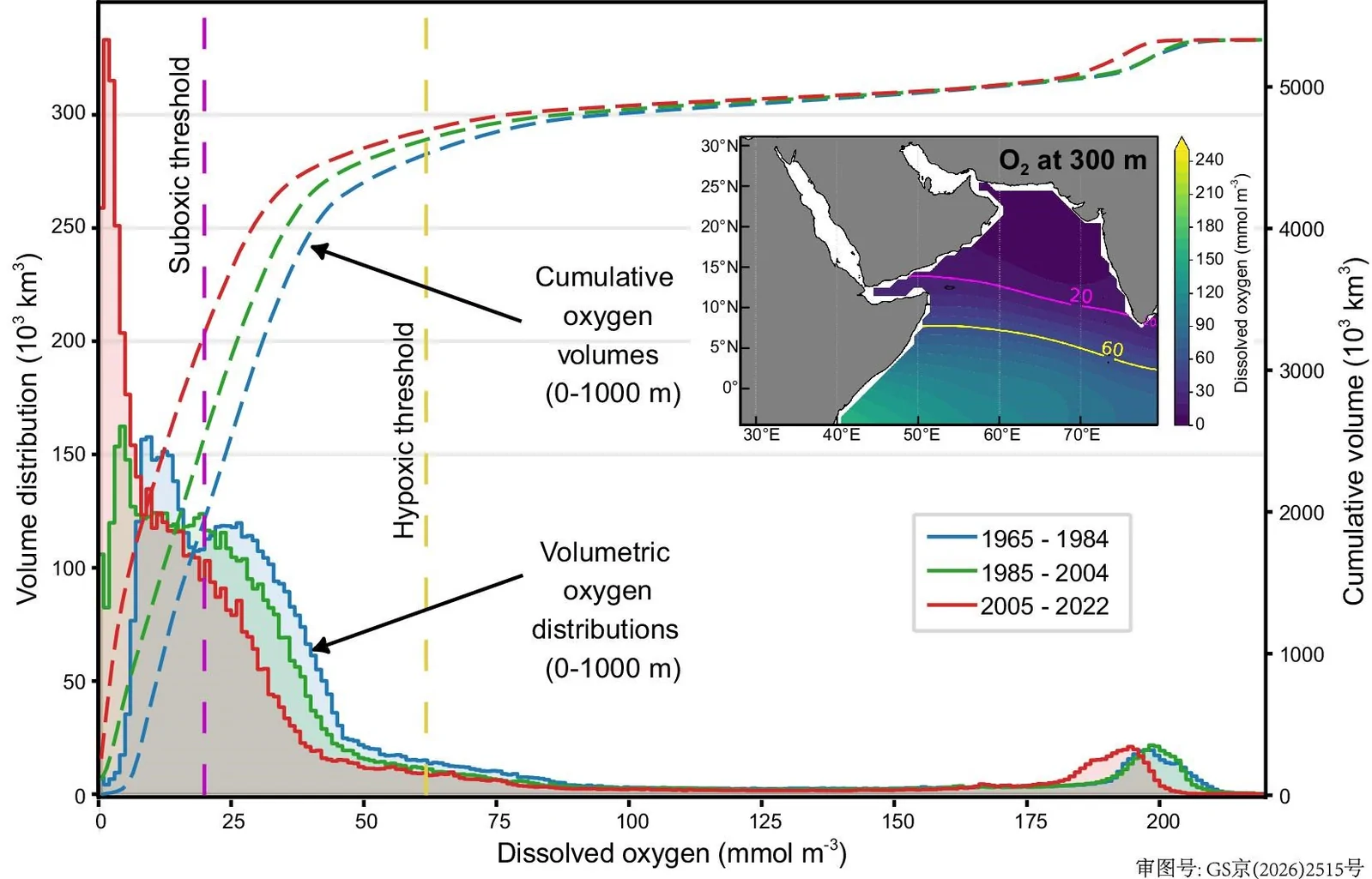
Evolution of the volumetric distribution of dissolved oxygen in the upper 1000 m of the Arabian Sea between 1965 and 2022. Solid curves and associated shading (left axis) show the volume of water within discrete oxygen concentration bins, while dashed curves (right axis) indicate cumulative volumes for three periods: 1965–1984, 1985–2004, and 2005–2022, as identified in the legend. Vertical reference lines indicate the thresholds for hypoxia (oxygen $< 63 \ \mu \rm {M}$) and suboxia (oxygen ${< }20 \ \mu \mathrm{M}$). The inset shows the horizontal distribution of dissolved oxygen at 300 m depth, with contours indicating the hypoxic and suboxic boundaries. The figure highlights a progressive expansion of the suboxic core of the oxygen minimum zone, accompanied by a reduction in the volume of hypoxic but non-suboxic waters ($20 \ \mu \rm {M} < $ oxygen $< 63 \ \mu \rm {M}$). Oxygen data are from [104].

Under recent climate forcing, historical observations and hindcast models reveal a severe deoxygenation trajectory. Between 1982 and 2010, $O_2$ inventories in the northern AS dropped by over 6% per decade in the 100–1000 m depth range [98]. This is due to surface warming that reduces the density of AGW outflows, causing the OMZ’s suboxic volume to expand at a rate of 14% per decade, leading to a 15% per decade increase in denitrification rates [98]. Critically, this deoxygenation is not confined to the open basin. The marginal seas, historically regarded as well-oxygenated refugia due to their shallow depths and vigorous mixing, are now exhibiting signs of systemic respiratory reduction [105]. In the AG, summer near-bottom oxygen levels now regularly fall to hypoxic levels, which is driven by a weakening of the Shamal winds, increasing the residence time of Gulf waters and allowing respiratory deficits to accumulate [28]. Similarly, in the RS, long-term monitoring has detected significant oxygen loss at mid-depths, and deep anoxic pools have recently been documented in the Farasan Banks [106]. These trends suggest that warming winters are suppressing the deep convection required to ventilate the deep RS, effectively severing the atmospheric lifeline to the deep ecosystem.

However, detecting the true trajectory of the OMZ is complicated by responses to physical forcing. Recent Biogeochemical-Argo float observations reveal a surprising recovery from deoxygenation within the core region of the AS OMZ since 2013, with average dissolved oxygen concentrations increased nearly threefold and the overall thickness of the OMZ decreased by 13% [107]. This oxygenation response has been linked to the weakening of the summer monsoon winds, which reduced coastal upwelling along the Oman coast, thereby decreasing primary production and subsequently lowering the biological oxygen demand at depth [107].

While observations and downscaled models show clear and rapid deoxygenation [108], ESMs often project weak changes or even re-oxygenation for the AS by 2100 [109]. This discrepancy likely arises from the models’ inability to resolve the fine-scale physical processes that govern the region’s oxygen budget. Addressing this uncertainty requires integrating high-resolution modeling with sustained observations to properly map the trajectory of this vital marine system. Meanwhile, declining ventilation and warming are central to basin-scale oxygen trends, whereas coastal hypoxia may be strongly modified by wastewater, nutrient loading, organic-matter enrichment, and restricted flushing. However, the relative contribution of these processes remains constrained outside intensively studied locations.

### Carbon cycling and ocean acidification

Carbon cycling in the Arabian Seas emerges from the close interaction between physical circulation, biological productivity, microbial remineralization, and oxygen dynamics. These interconnected processes govern the efficiency of the biological carbon pump (BCP), regulate carbon sequestration, air–sea CO$_2$ exchange, and the evolution of ocean acidification, thereby underpinning the ocean’s role in the global carbon cycle [110].

In the open AS, the efficiency of the BCP is governed by the strong coupling between monsoon-driven primary production and the export of organic carbon to depth. While the southwest monsoon upwelling fuels intense phytoplankton blooms, the efficiency of transferring this organic carbon to the deep ocean is increasingly non-linear [111]. Ocean warming enhances heterotrophic metabolic rates in the upper ocean [112], accelerating the remineralization of sinking organic matter before it reaches depth and thereby reducing long-term carbon sequestration [110]. Consequently, carbon export is controlled not only by primary production but also by the efficiency of microbial remineralization and grazing within the upper water column, which regulate the fraction of organic carbon that escapes to the deep ocean [113].

Importantly, these dynamics intersect with the OMZ described in the preceding section. As oxygen concentrations decline, microbial pathways involved in organic matter remineralization shift, altering the fate of exported carbon. Within the suboxic core of the OMZ, the suppression of zooplankton grazing and changes in microbial activity can enhance the preservation of sinking particles [114]. This suggests a feedback loop where the expansion of OMZ temporarily mitigates the loss of carbon export efficiency, altering the attenuation of particulate organic carbon (POC) flux in ways that current global models often fail to capture [114]. Such interactions remain insufficiently represented in many global carbon cycle models.

In contrast, the marginal seas provide analogs for carbon cycling in future ocean states characterized by extreme stratification (RS) and anthropogenic eutrophication (AG). The RS illustrates how strong thermal stratification and oligotrophic conditions shape carbon cycling and the efficiency of the BCP. Here, carbon export is sustained primarily by episodic processes, including mesoscale eddies, winter mixing, and localized nutrient injections, rather than by persistent seasonal blooms [115]. However, the basin’s persistently warm waters enhance remineralization rates, such that only a small fraction ($\sim 10\%$) of exported POC survives transit through the upper mesopelagic zone, limiting long-term carbon sequestration [100]. In this oligotrophic environment, carbon cycling increasingly relies on the dissolved organic carbon (DOC) pathway, whereby the production and subduction of semi-labile DOC contribute substantially to carbon export and sustain microbial metabolism below the euphotic zone in addition to POC export [116]. Conversely, the AG represents a marine system in which human perturbations overwhelm natural cycles. Extensive eutrophication has elevated total organic carbon (TOC) loads in surface waters. As this excess organic matter sinks and is remineralized, microbial respiration consumes dissolved oxygen while releasing $\mathrm{CO_{2}}$, thereby accelerating acidification alongside hypoxia [117]. Modeling studies further suggest that the weakening of the Shamal winds exacerbates these processes by reducing ventilation, trapping warm, carbon-rich waters within the basin, and modifying carbon cycling and biogeochemical budgets [28].

The carbon cycle strongly influences ocean acidification. At the regional scale, the Arabian Seas are accumulating anthropogenic carbon, in addition to the CO$_2$ generated through organic matter remineralization, accelerating the progression of ocean acidification. The region is naturally predisposed to high inorganic carbon, acting as a source of $\mathrm{CO_{2}}$ to the atmosphere due to monsoon-driven outgassing [118]. However, acidification is driven and intensified by the combined effects of rising atmospheric $\mathrm{CO_{2}}$ and the local accumulation of dissolved inorganic carbon (DIC) from remineralization [119]. These processes are reflected at the basin scale, where the Indian Ocean experienced a decline in surface pH of *∼*0.0135/decade, slightly lower than the global ocean average (*∼*0.018/decade) [119]. The increase in DIC was the dominant driver, accounting for nearly 69.3% of the acidification trend, while seawater warming contributed *∼*13.8% [119]. Because a consistent pre-industrial baseline for aragonite saturation state ($\Omega _{{\rm arag}}$) is not available across the Arabian Seas, it is currently not possible to assess whether the Ocean Acidification PB has been regionally transgressed. Instead, pH and DIC are used here as regional indicators of acidification pressure.

This acidification poses a threat to Biosphere Integrity, particularly concerning biogenic calcifiers in the AG and RS. In the AG, organisms are forced to accrete calcium carbonate skeletons against a backdrop of both extreme, near-lethal thermal stress and rapidly dissolving chemical conditions. Furthermore, coastal Blue Carbon ecosystems, including mangroves, seagrasses, and saltmarshes, which typically provide a crucial buffer by sequestering carbon, are having their functional integrity severely compromised by acidification [120]. In the heavily industrialized AG, extreme coastal development, hypersalinity, and high soil respiration rates have worsened the acidification-influenced physiological capacity of these ecosystems [121].

Overall, the Arabian Seas illustrate how global climate change and regional anthropogenic pressures interact to modify marine carbon cycling and accelerate ocean acidification. They demonstrate that as the physical limits of the climate system are crossed, the BCP’s ability to regulate the ocean’s chemical state could be compromised [122]. When rising CO$_{2}$ is amplified by regional anthropogenic eutrophication and temperature-driven shallow remineralization, local pH and aragonite saturation may decline more rapidly. In particular, in eutrophic coastal waters, respiration of wastewater- or productivity-derived organic matter may generate additional short-term and seasonal pH and aragonite-saturation declines. This reinforces the necessity to investigate regional indicators of increasing ecological risk.

## MARINE ECOSYSTEM REORGANIZATION

The physical amplifiers and biogeochemical consequences described in the preceding sections jointly drive biological responses and regime shifts. Direct anthropogenic pressures also reduce resistance to those stresses and slow recovery after disturbance. As the Earth transgresses PBs, resident ecosystems may increasingly lose their capacity to buffer those extremes. This section synthesizes the functional responses of marine ecosystems to this multi-stressor environment.

### Coral refugia and adaptation

The coral reefs in the Arabian Seas serve as a critical natural laboratory for understanding the limits of coral resilience in a warming world. The contrasting responses of the RS and the AG illustrate two distinct trajectories for tropical reefs under ongoing climate change: the persistence of a climate refuge and the emergence of highly stress-tolerant coral communities.

In the northern RS, coral communities exhibit a marked decoupling from the global thermal bleaching paradigm (Fig. 6). Corals here persist at summer maxima of ${\sim }27^{\circ }$C while exhibiting no signs of bleaching when exposed to experimental or anomalous in situ temperatures up to 32–34$^{\circ }$C, maintaining a physiological safety margin of +5 to $+7^{\circ }$C [6,123]. This resilience is attributed to a historical thermal filter mechanism. Genetic and physiological evidence suggest that extant populations descend from lineages that migrated through the significantly warmer southern RS during the post-glacial recolonization [6]. This evolutionary process likely pre-selected heat-resistant genotypes, endowing northern populations with a thermal tolerance that exceeds their current environmental ambient maxima [123].

**Figure 6.**
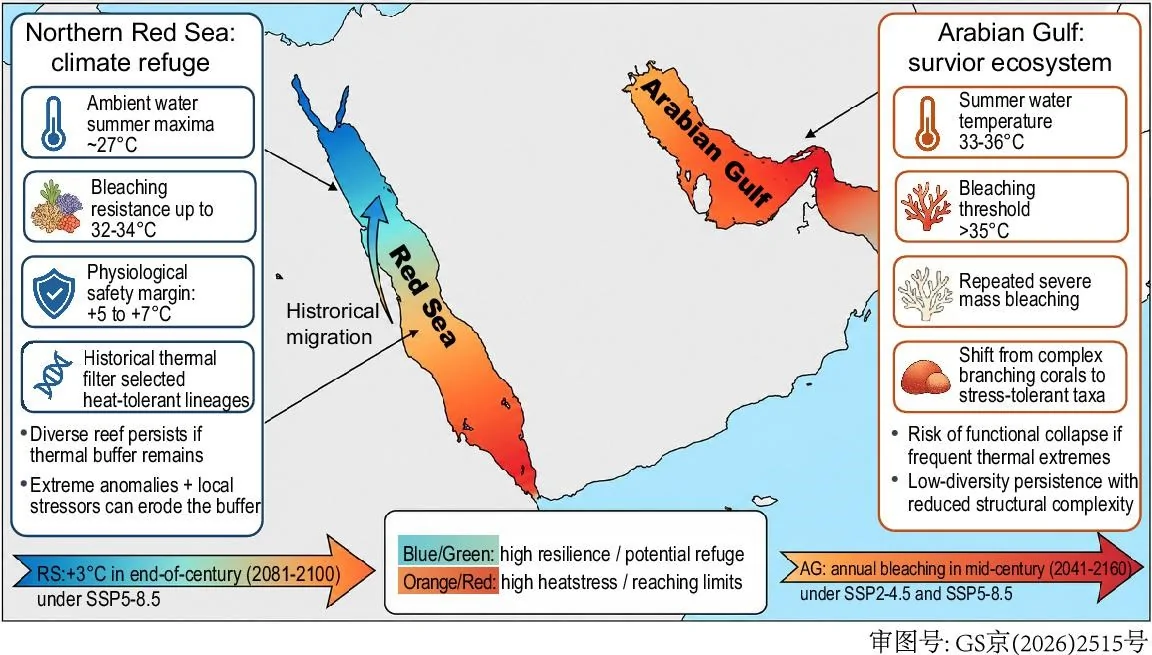
Schematic representation of heat tolerance and projected climate limits in Arabian region coral reefs. The map illustrates contrasting thermal regimes and resilience profiles across the Red Sea (RS) and Arabian Gulf (AG), distinguishing areas with high potential thermal resilience from those experiencing intense heat stress and approaching physiological thermal limits. Bottom annotations indicate future climate projections. By the end of the century (2081–2100), high-emissions scenarios (SSP5-8.5) project regional warming of up to $3^{\circ }$C in the northern RS, potentially reducing the physiological safety margin that currently supports its role as a climate refuge. In the AG, under both SSP2-4.5 and SSP5-8.5 pathways, extreme summer sea surface temperatures are projected to routinely exceed critical bleaching thresholds by mid-century (2041–2060). These projections suggest that even highly heat-tolerant coral ecosystems may face increasing risks of exceeding their thermal limits under continued warming.

Consequently, the northern RS is widely regarded as a climate refuge (Fig. 7a). Under high-emissions scenarios (SSP5-8.5), model projections indicate that regional SST may rise by $3^{\circ }$C by the end of the century (2081–2100) yet remain below the bleaching threshold, making it one of the most promising potential refugia for coral biodiversity when other tropical basins are no longer viable [6,25,124]. However, this resilience has clear limits. The extreme thermal anomalies associated with the super-El Niño events of recent years have begun to erode this buffer (Fig. 7b and c). Bleaching events recorded in the central RS during 2015 demonstrate that when the Climate Change PB interacts with local stressors (Fig. 7d), even these robust communities can experience mortality [125]. The long-term persistence of this refuge will depend on mitigating local stresses, such as eutrophication and coastal development that compromise the physiological headroom of the corals [126].

**Figure 7.**
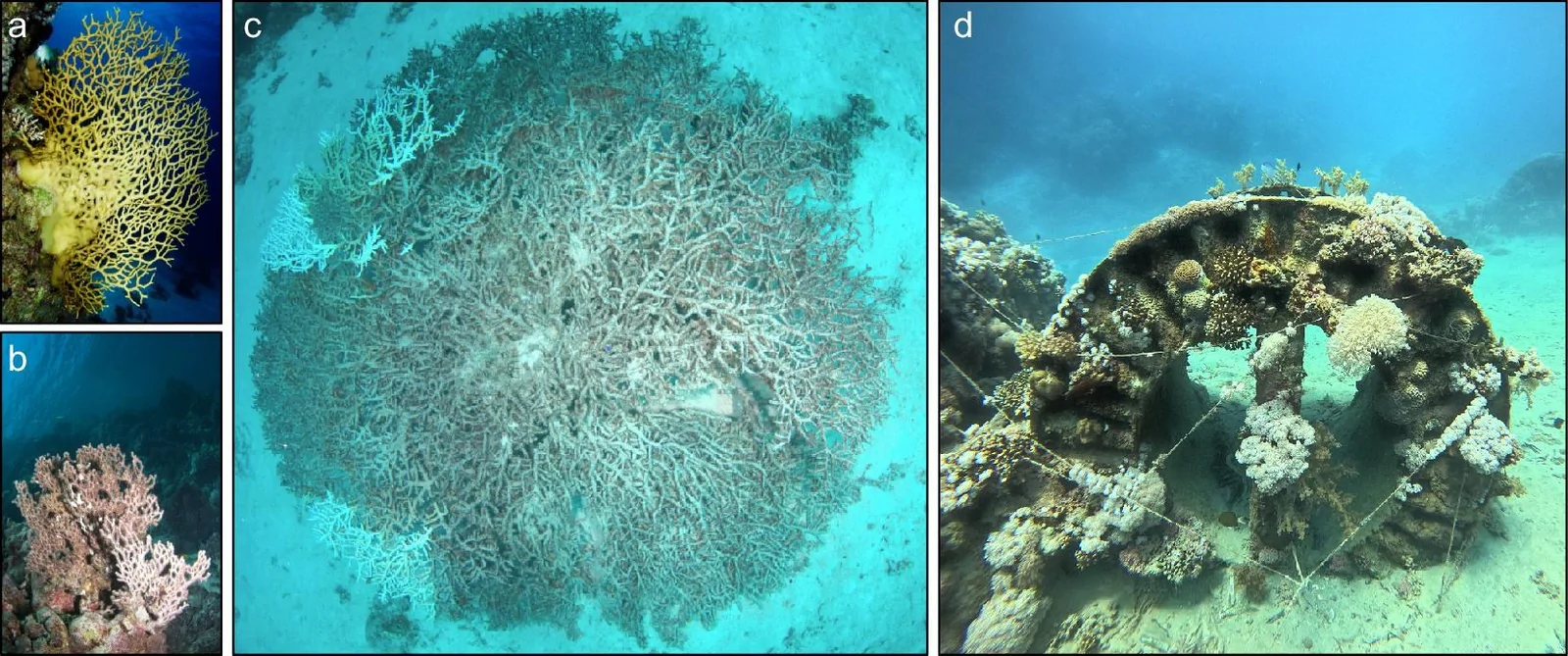
Corals in the RS. (a) A healthy, living colony of the fire coral *Millepora dichotoma*. (b) A dead colony of *Millepora dichotoma*, exhibiting loss of tissue and algal colonization. (c) A large colony of *Acropora pharaonis* displaying partial mortality and bleaching; the white outer margins consist of living but bleached tissue, contrasting with the dark gray central portion, which represents the exposed skeleton of dead coral. (d) A diverse coral community colonizing a discarded metal structure entangled with fishing lines. Together, these scenes capture the dual reality of the Northern RS: While the ecosystem exhibits extraordinary hardiness to persist amidst direct local stressors (e.g. marine debris), the compounding pressures of global climate extremes and local anthropogenic interference are actively testing the limits of this critical climatic refuge.

The AG represents an ecosystem that has adapted to some of the warmest conditions on Earth (Fig. 6). Here, corals exist near their upper thermal limits of calcification, routinely enduring summer temperatures of 33–$36^{\circ }$C lethal to most corals worldwide [127]. The bleaching threshold in the AG is high, requiring exposure to temperatures $> 35^{\circ }$C for eight weeks to trigger mortality [128]. Nevertheless, although coral thermal tolerance is heritable [129], whether adaptive responses can keep pace with rapid ocean warming remains uncertain [130]. Realized adaptation may be constrained by generation times, demographic decline, genetic trade-offs, and holobiont interactions, limiting the efficacy of standing genetic variation under climate change [126]. Recent monitoring across ten reef sites spanning *∼*350 km in Abu Dhabi revealed repeated severe bleaching events, as SST exceeded the local ecological threshold for coral bleaching and mortality [128]. Hard-coral cover and fleshy macroalgal cover declined by 78% and 83% from 2010 to 2020, respectively, whereas turf algae and coralline algae increased, resulting in a degraded but non-macroalgal-dominated benthic state [128].

Even when surviving from bleaching events, this resilience comes at an ecological cost, which eliminates fast-growing and structurally complex branching taxa (e.g. *Acropora*) and favors a simplified community dominated by slow-growing and stress-tolerant genera (e.g. *Porites*) [131]. As a result, the ecosystem functional diversity and topographic complexity are reduced. The AG therefore provides insights into how coral reef communities may behave under continued warming, with a system adapted to extremes through biotic homogenization and the loss of structural integrity. Climate projections under both intermediate (SSP2-4.5) and high-emissions (SSP5-8.5) pathways suggest that by mid-century (2041–2060), the frequency of thermal extremes in the AG could become annual [132], potentially exceeding even the adapted limits of these super corals and pushing the system from a state of low-diversity persistence toward functional degradation [130].

Spatial and taxonomic variation in thermal tolerance across the RS are reported, but the extent to which this variation reflects host genetics, symbiont identity, acclimatization, or their interaction remains incompletely resolved [133]. Whether the observed thermal tolerance is sufficiently heritable and evolvable to track the pace of projected warming remains an open question. It is worth noting that the high resilience of these marginal sea corals is contingent upon the region’s specific evolutionary processes and its exceptionally high total alkalinity (*∼*2400 *μ*mol kg$^{-1}$), which acts as a powerful chemical buffer mitigating direct localized pressures resulting from the ocean acidification [134]. Open-ocean tropical reefs lack this specific genetic history and robust chemical buffering capacity; therefore, they may not be expected to naturally replicate this degree of thermal resilience. The Arabian Seas not only demonstrate the absolute physiological ceiling of coral adaptation, but also signal that global open-ocean reefs will cross critical thermal thresholds much earlier.

### Pelagic regime shifts

In the open AS, increasing stratification, altered nutrient stoichiometry, and shoaling low-oxygen waters jointly favor a shift from diatom-dominated production toward *Noctiluca*-dominated assemblages [135]. The precise drivers of this ecological shift, however, remain controversial, highlighting the complexities of response to global PB transgressions at the regional scale. One hypothesis links *Noctiluca* outbreaks to the regional expansion and shoaling of the AS OMZ through its effects on oxygen availability and nitrogen cycling [136]. An influx of hypoxic waters into the lower euphotic zone provides a distinct competitive advantage to *N. scintillans*, whose endosymbiont *Pedinomonas noctilucae* is uniquely adapted to fix carbon efficiently under severe oxygen deficiency, while diatom photosynthesis concurrently drops by ${\sim }50\%$ [97].

However, this hypothesis is debated by recent observational studies. They refute the presence of surface hypoxia and demonstrate that dissolved oxygen in the photic zone consistently remains ${> }70\%$ saturated during *Noctiluca* outbreaks [137] in the northeastern AS. This opposing framework posits that the regime shift is likely driven by a stoichiometric bottleneck caused by physical stratification. Historically, the winter northeast monsoon drove convective mixing that supplied silicate and nitrate to the euphotic zone, fueling diatom blooms that underpinned the region’s rich fisheries. The rapid warming of the Eurasian landmass has weakened the winter monsoon winds, while upper-ocean warming has intensified stratification and reduced convective mixing [27,138]. Because the silicocline is located deeper than the nitrocline in the AS, this physically weakened vertical mixing disproportionately changes the upward flux of silicate relative to nitrate. Diatoms, which require high concentrations of silicate for frustule formation, face severe nutrient limitation [137]. *N. scintillans*, unconstrained by silicate requirements and highly capable of phagotrophy, effortlessly outcompetes diatoms in this newly stratified, stoichiometrically altered environment [139].

Regardless of the precise biogeochemical trigger, the ecological consequence is a highly damaging trophic cascade that changes the transfer of energy to higher trophic levels. The large size of *N. scintillans* prevents efficient grazing by traditional mesozooplankton, leading to a trophic dead-end for the traditional crustacean-fish food chain [97]. Instead, these blooms support a proliferation of gelatinous zooplankton such as salps and jellyfish that can feed on *Noctiluca* [27]. As a result, the energy that historically flowed into commercial fish stocks is now shifted into a gelatinous biomass pathway [140]. The gelatinous web promotes rapid surface recycling and alters the sequestration capacity of the basin. Meanwhile, this shift signals a large reduction in the carrying capacity for harvestable marine resources, replacing a protein-rich food web with a localized ecosystem that offers little utility to human society and could potentially disrupt coastal infrastructure [141]. Recovery toward the former diatom-dominated state would likely require a substantial reduction in the warming- and stratification-related pressures, and may also depend on restoration of nutrient stoichiometry and oxygen conditions. However, the stability and reversibility of this community reorganization have not been tested directly.

### Physiological constraints

The stress experienced in the Arabian Seas extends beyond shifts in community composition to the physiological limits of individual organisms.

Among all life-history stages, the reproductive phases of fishes appear to be the most vulnerable to climate stress. The development of fish eggs and larvae is strongly temperature-dependent [142]. Higher temperature accelerates embryonic development, therefore increasing metabolic oxygen demand. Meanwhile, warmer water holds less dissolved oxygen, potentially resulting in a mismatch between oxygen supply and demand [143]. This physiological squeeze forces embryos to rapidly deplete yolk reserves, resulting in earlier hatching at smaller sizes and with reduced energetic capital [144]. Consequently, the recruitment success of many reef fishes is increasingly confined to cooler seasonal windows. Observations from the RS support this mechanism, showing that peak recruitment for many reef fishes is now confined to cooler seasonal windows that avoid the summer thermal maximum [145]. As summer warming intensifies and lengthens, this window is likely to shrink. Furthermore, the combined pressure of warming and deoxygenation is also driving a reduction in the average body size of adult fishes across the region [146]. In the AG, adult reef fishes, ranging from cryptobenthic species to large piscivores, are 14%–40% smaller at maximum size compared to conspecific populations inhabiting cooler environments [147].

It is worth noting that other factors, such as fishing pressure, degradation of nursery habitats, and food web changes, could also cause size reduction, although their relative contributions have not been quantified consistently across species and basins [148]. Fishing pressure exacerbates size reduction by artificially truncating age distributions and evolutionarily selecting for earlier sexual maturation at smaller sizes [149]. Therefore, direct fishing pressure acts as a synergistic accelerator, leading to a severe trophic amplification of biomass declines [150]. Although fishing can exacerbate reductions in observed population size structure, controlled physiological studies indicate that elevated temperature can constrain growth and maximum attainable size independently of direct size-selective harvest [131]. However, the relative contributions of warming and fishing to observed field populations remain unresolved.

The physiological mechanisms underlying this size reduction have been debated. The prevailing Gill-Oxygen Limitation Theory (GOLT) posits that fish body size is limited by the geometric constraints of gill surface area [151]. A second hypothesis, Maintain Aerobic Scope and Regulate Oxygen Supply (MASROS), suggests that developmental plasticity restricts body size at elevated temperatures to maintain a critical safety margin of aerobic scope needed for swimming and digestion [131]. However, respirometry and kinematic studies in the AG refute the universality of these theories, and, instead, they suggest that an energy acquisition–demand mismatch may be more important than a simple limitation of oxygen uptake [131]. While these fishes can extract enough oxygen, larger individuals cannot linearly scale their maximum prey capture and digestive efficiencies to meet this metabolic requirement [152]. To offset this, smaller body sizes are favored as observed in wild AG populations [131], allowing them to remain within the energetic acquisition limits of their environment. This physiological downsizing propagates upward through the food web, altering predator–prey interactions and weakening top-down control [150]. Such organism-level responses may propagate through population and food-web processes, but whether they generate a discrete ecosystem-level threshold, and where such a threshold might occur remains to be further investigated.

## LESSONS FROM AN OCEAN AT THE EDGE

The Arabian Seas offer a natural laboratory for examining the future trajectory of marine ecosystems under anthropogenic forcing. Across this review, we systematically evaluate the regional responses to the transgression of multiple global PBs. Figure 8 synthesizes the resulting causal framework, illustrating how global environmental forcing and regional anthropogenic pressures interact through physical amplification and biogeochemical changes to drive ecological reorganization in the Arabian Seas. The regional observations provide transferable insights into mechanisms of resilience loss, recovery constraints, and ecological reorganization that may become increasingly relevant in other tropical and marginal marine systems.

**Figure 8.**
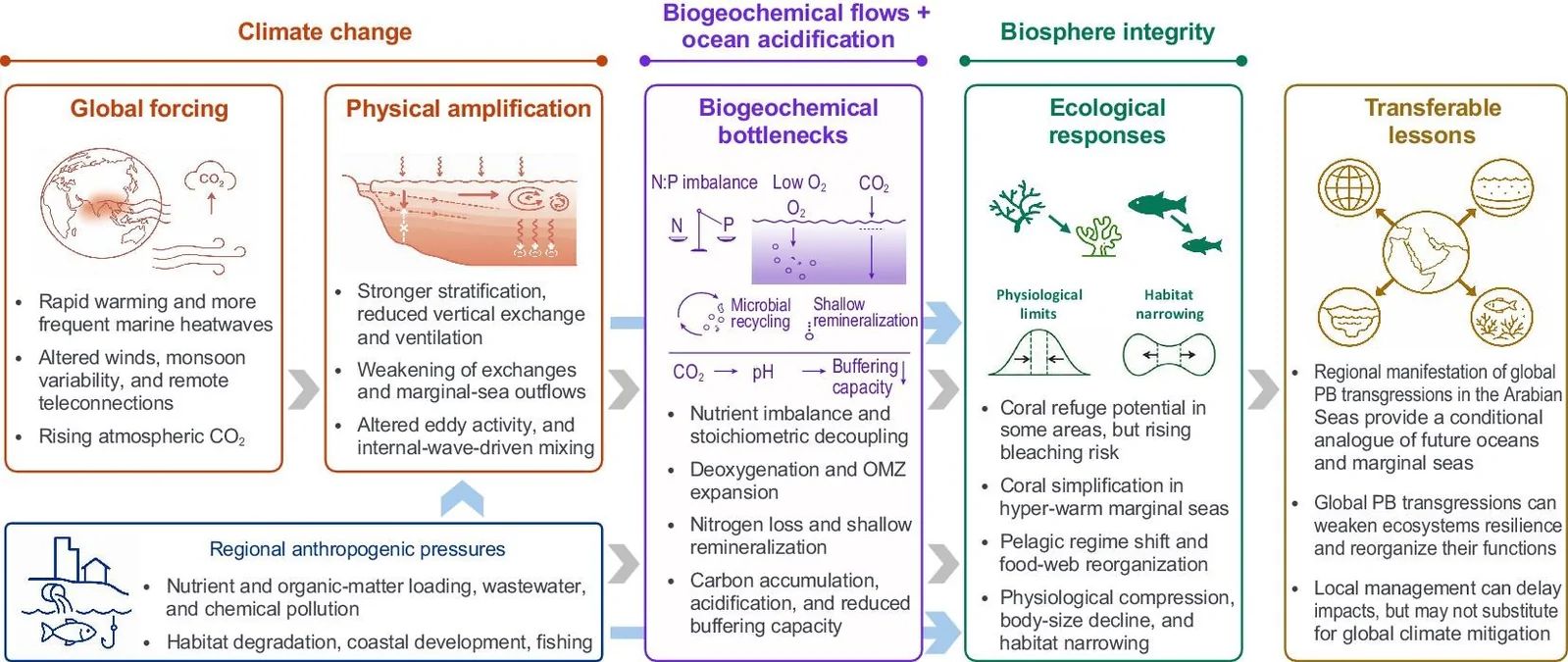
Conceptual synthesis of the review’s central causal chain: from planetary forcing to ecosystem reorganization in the Arabian Seas. The headings denote the four global PBs assessed in this review: Climate Change, Biogeochemical Flows, Ocean Acidification, and Biosphere Integrity. Additionally, regional anthropogenic pressures are integrated into the framework, demonstrating how direct local human activities synergistically exacerbate these regional environmental shifts. The sequential boxes below depict the regional manifestations and cascading impacts of these PBs across the Arabian Seas, mapping the pathway from global forcing and physical amplification to biogeochemical bottlenecks, subsequent ecological responses, and transferable lessons.

### Regional responses and remaining controversies

The PB framework was formulated to quantify aggregated interferences at the global Earth system scale, and the regional transitions suggest the potential pathways through which regional ecosystem integrity could degrade. We do not frame the Arabian Seas as a full future ocean analogy. For instance, the biogeochemical and ecological extremes observed in the AG and the RS are governed by their unique geometric and hydrographic constraints. Consequently, these marginal basins do not represent the future average conditions of the global open ocean. Instead, they serve as specific analogs for the extreme thermodynamic and hypersaline conditions that will increasingly characterize future marginal seas. While certain trajectories observed here represent local phenomena, the broader mechanisms, such as stratification-induced deep-water ventilation weakening, the stoichiometric decoupling of pelagic food webs, and the erosion of carbonate buffering capacity, provide highly transferable lessons. By accelerating the timeline of climate stressors, the Arabian Seas function as a sentinel system for investigating mechanisms through which marine ecosystems may reorganize under intensifying planetary forcing.

Evidence from the Arabian Seas documents ecological reorganizations under multiple stressors. Although these changes may indicate declining resilience and increasing risk of transitions, the available observations generally do not establish tipping points in the formal sense because hysteresis, alternative stable states, and self-sustaining feedbacks have rarely been tested. Additionally, our capacity to project these responses is often hindered by diverging model projections and unresolved scientific controversies. Table 1 summarizes this state of knowledge for the four (PBs), offering a structured assessment of established mechanisms alongside unresolved attribution issues.

**Table 1.** Regional manifestations and analytical interpretation of pressures associated with four global PBs in the Arabian Seas. In the PB framework, each global boundary is assessed using one or more control variables that describe the state of a critical Earth-system process. Regional manifestations are used as indicators of how pressures associated with PB change are expressed and amplified within the Arabian Seas, not substitutes for the global PB control variables.

Earth system process	Global PB representation	Regional manifestation	Principal contributing factors	Competing interpretations or projections	Critical knowledge gap
Climate change	Perturbation of the global climate system, evaluated using global control variables	SST warming, marine heatwaves, stratification, altered winds and circulation, weakening marginal-sea outflows, and declining ventilation	Anthropogenic forcing, ENSO, IOD, AMO, NAO, local winds, freshwater anomalies	Uncertainty in Pacific mean-state change and associated teleconnection responses	Narrow gateways and ventilation pathways remain poorly represented
Biogeochemical flows	Global disruption of nitrogen and phosphorus cycles	Nutrient loading, altered nutrient stoichiometry, denitrification, nitrogen loss, OMZ expansion, and eutrophication	Ventilation, export production, respiration, marginal-sea outflows, eutrophication	OMZ expansion versus contraction or temporary reoxygenation	The balance between physical oxygen supply and biological oxygen demand remains insufficiently constrained
Ocean acidification	Global change in ocean carbonate chemistry, defined using surface-ocean aragonite saturation relative to its preindustrial state	Decreasing pH, DIC, remineralization, and available $\Omega _{\mathrm{arag}}$ estimates	Atmospheric CO$_2$, organic-matter remineralization, eutrophication, warming, circulation, wastewater-derived respiration	Relative contributions of external CO$_2$ loading and local respiratory acidification	Consistent preindustrial $\Omega _{\mathrm{arag}}$ baselines are unavailable for entire basin
Biosphere integrity	Loss of genetic and functional integrity of the biosphere	Coral-community change, plankton regime shifts, fish recruitment and size, trophic structure, and functional diversity	Thermal stress, acclimatization, hypoxia, nutrients, fishing, food-web change, pollution, coastal development	Adaptation versus acclimatization; hypoxia versus nutrient limitation; ecosystem persistence versus functional collapse	Causal attribution remains limited, and multi-generational observations are scarce

In the open AS, the future trajectory of the OMZ remains a subject of debate. Coarse ESMs are inadequate for resolving fine-scale physical ventilation mechanisms and may therefore fail to predict physical-driven processes relevant to the Biogeochemical Flows PB. This limitation fuels controversies regarding the mechanisms of the regime shift in the pelagic ecosystem, introducing uncertainties in response to the transgression of global Biosphere Integrity PB. In the benthic realm, the survival of coral reefs in the RS and AG illustrates divergent outcomes of thermal adaptation. Global open-ocean reefs, which generally lack the evolutionary history and chemical buffering capacity found in the RS and the AG, will likely undergo similar reorganizations at lower thermal thresholds. Nevertheless, the exact trajectory remains uncertain in the absence of detailed, site-specific surveys.

### Adaptive strategies and future outlook

To navigate the safe operating space of the Anthropocene, the scientific and management communities must move beyond identifying isolated environmental stressors toward implementing adaptive strategies to mitigate the impacts of multiple global PB transgressions. Although regional management cannot reverse global climate forcing, reducing direct local anthropogenic pressures can lessen regional impacts, enhance ecological resilience, and increase opportunities for acclimatization or adaptation.

First, comprehensive observational networks should be expanded to continuously monitor hydrodynamic choke points and coastal margins. Water mass exchanges through the Straits of Hormuz and Bab-el-Mandeb strongly influence nutrient and oxygen budgets of the interior basins. Long-term, high-frequency monitoring utilizing autonomous instruments and advanced remote sensing will be essential for resolving these fine-scale processes. Additionally, monitoring changes in the frequency, duration, and intensity of MHWs is essential for understanding cumulative physiological stress. As MHW frequency increases, the window for ecological recovery narrows. Identifying the temporal evolution of these shifts is therefore fundamental to predictive ecological management.

Second, the development of high-resolution regional ocean reanalyses and coupled modeling systems is critical. Current global ESMs are often too coarse to resolve the submesoscale frontal dynamics and marginal sea overflows that mediate regional biogeochemical processes. Initiatives such as the RS Regional Reanalysis (RSRA), which utilizes 1-km to 5-km grid resolutions and data assimilation, provide a suitable framework for resolving seasonal overturning anomalies, eddy-induced tracer transport, and shifting thermal limits [153]. Expanding these high-resolution, coupled physical-biogeochemical models across the Arabian Seas will enable more reliable forecasts of hypoxia expansion, nutrient stoichiometry shifts, and the dispersion pathways of marine larvae.

Third, strengthening transboundary governance will be essential to mitigate localized stressors. Particularly in marginal seas, the impacts of climate change are amplified by direct human activities. Establishing transboundary Marine Protected Areas (MPAs) could help protect upstream refugia of heat-resilient genotypes capable of reseeding degraded downstream habitats. Furthermore, mitigating transboundary anthropogenic eutrophication and chemical pollution is essential to prevent the localized exacerbation of the Ocean Acidification and Biogeochemical Flows PBs. Local intervention cannot reverse global forcing, but it can substantially reduce regional vulnerability and improve ecosystem resilience.

A key insight from this review is that the transgression of PBs may progressively reduce the resilience of marine ecosystems. Whether these adapted states are dynamically stable, reversible, or separated by hysteresis remains to be investigated. In addition, some regional pressures, particularly synthetic pollutants, hydrocarbons, and desalination discharges, are not represented fully by the four PBs emphasized here and may intersect with the Novel Entities and Freshwater Change PBs. Their exclusion from the primary review structure does not imply ecological insignificance. Future research should focus on quantifying the proximity to these processes, integrating physiological limits with community-level interactions. This knowledge is valuable for developing management strategies and will offer a vital toolkit for navigating the complex changes facing the global ocean in the Anthropocene.
